# A Review of the Methods for Detection of *Staphylococcus aureus* Enterotoxins

**DOI:** 10.3390/toxins8070176

**Published:** 2016-06-24

**Authors:** Shijia Wu, Nuo Duan, Huajie Gu, Liling Hao, Hua Ye, Wenhui Gong, Zhouping Wang

**Affiliations:** State Key Laboratory of Food Science and Technology, Synergetic Innovation Center of Food Safety and Nutrition, School of Food Science and Technology, Jiangnan University, Wuxi 214122, China; wusj1986@163.com (S.W.); duannuo@jiangnan.edu.cn (N.D.); liongu@mail.usts.edu.cn (H.G.); hll1111@126.com (L.H.); yehua_2004@163.com (H.Y.); gongwhuo15@163.com (W.G.)

**Keywords:** Staphylococcal enterotoxins, food-borne poisoning, detection, analysis

## Abstract

Food safety has attracted extensive attention around the world, and food-borne diseases have become one of the major threats to health. *Staphylococcus aureus* is a major food-borne pathogen worldwide and a frequent contaminant of foodstuffs. Staphylococcal enterotoxins (SEs) produced by some *S. aureus* strains will lead to staphylococcal food poisoning (SFP) outbreaks. The most common symptoms caused by ingestion of SEs within food are nausea, vomiting, diarrhea and cramps. Children will suffer SFP by ingesting as little as 100 ng of SEs, and only a few micrograms of SEs are enough to cause SPF in vulnerable populations. Therefore, it is a great challenge and of urgent need to detect and identify SEs rapidly and accurately for governmental and non-governmental agencies, including the military, public health departments, and health care facilities. Herein, an overview of SE detection has been provided through a comprehensive literature survey.

## 1. Introduction

Currently, food-borne disease caused by bacterial contamination is one of the biggest issues affecting human health and food safety. From 2011 to 2014, 4211 foodborne disease outbreaks were reported in China. Of the 3183 outbreaks with a known etiologic agent, pathogenic microorganisms were confirmed in 1244 (39%), which resulted in 27,479 illnesses [1,2]. Among these illnesses, *Staphylococcus aureus* was recognized as one of the most prominent culprits, resulting in 3269 illnesses (11.9%) [1,2]. *S. aureus* is a Gram-positive microorganism that colonizes the nasal passages and skin of approximately 50% of healthy individuals. *S. aureus* grows in a wide range of temperatures and pH, from 7 °C to 48.5 °C, and 4.2 to 9.3, respectively. *S. aureus* can adapt to grow in various foods and causes food poisoning by secreting enterotoxins [3].

Staphylococcal enterotoxins (SEs) are members of a family of more than 20 different staphylococcal and streptococcal exotoxins, sharing a common phylogenetic relationship, structure, function, and sequence homology. Presently, 23 enterotoxins have been identified as distinct serological entities [4], including SEA, SEB, SEC, SED, and SEE. These toxins are basic proteins made up of approximately 220–240 amino acids and have similar molecular weights of 25–30 kDa. The most common SEs are SEA and SEB. SEA is most frequently involved in food poisoning caused by staphylococcus [5]. SEB is not only involved in food poisoning but identified as a potential biological weapon of war and terrorism [6]. Various foods can be contaminated by SEs, especially moist foods containing starch and protein, such as meat and meat products, poultry and egg products, and milk and other dairy products. Most of the SFP outbreaks are due to improper food handling either in the food industry or in the home. Few outbreaks can be traced directly to contamination during food processing. Production of SEs rapidly increases at suitable temperatures (20–37 °C) and pH (4–7.4) [7,8]. The most common symptoms resulting from the ingestion of food contaminated by SEs are nausea, vomiting, diarrhea and abdominal cramps, which occur within 2–6 h of eating SE-contaminated food [9,10]. The mechanism of SE-induced food poisoning is still not fully understood. Some researchers have produced evidence that SFP results in an inflammatory response throughout the gastrointestinal tract, characterized by serious damage in the jejunum and ileum [10]. Other researchers have shown that SEs do not directly act on the gastrointestinal tract, but indirectly affect the expression of cytokines and metabolites produced by T cells, macrophages, monocytes and mastocytes [11,12]. Children will suffer SFP by ingesting as little as 100 ng of SEs, and vulnerable populations may develop staphylococcal food poisoning with a few micrograms of toxin [13,14]. In addition, SEB has been identified as a restricted agent by the Centers for Disease Control and Prevention (CDC) [15]. A very small amount of enterotoxin will cause intoxication, and the LD_50_ of SEB in monkeys is 0.02 μg/kg of body weight [16].

As a result, SEs are a threat to both food safety and food security if they are produced in a purified form that can be used as a deliberate adulterant. Consequently, it is crucial to develop reliable, sensitive, and rapid methods for the detection of SEs. A large number of sensitive and selective detection methods based on different principles have been reported. This review provides a brief overview of conventional methods and focuses on immunosensors, which are currently used to detect SEs in food. Finally, future trends and conclusions are discussed.

## 2. SE Identification Using Conventional Methods

### 2.1. Animal Tests

Animal tests were among the earliest methods developed to detect SEs and include kitten and monkey studies. SEs are fed to animals, and the resultant physiological changes, such as vomiting and diarrhea, are studied. As early as 1936, Dolman, Wilson and Cockcroft introduced the kitten test for the detection of enterotoxins in staphylococcal filtrates [17]. The test has been widely used in public health and in the fundamental study of the nature of enterotoxins. However, this test has low sensitivity and specificity. Thus, a direct skin test was developed by using highly sensitized guinea pigs to detect SEB in food, such as chicken meat and salami [18]. The assay could measure 10–100 pg of SEB per mL of prepared food sample, and the entire process required less than 20 min. However, the other SEs failed to produce a similar skin response to that of SEB. The practicability was so poor that the method has not been widely applied. Overall, the use of animal tests are restricted as a result of difficulties in animal origin and individual differences. Because of these shortcomings, along with the expense of animals, this method for the detection of SEs has fallen into desuetude.

### 2.2. Serologic Tests

Serologic tests are some of the earlier methods applied for SEs’ detection, including the gel diffusion test and agglutination test. These tests are essentially reactions based on antigen/antibody binding. Because antibodies mainly exist in serum, *in vitro* antigen/antibody reactions have been regarded as serologic tests characterized by precipitation and agglutination reactions. The gel diffusion test is based on the precipitation produced by antigen/antibody interaction, whereas the agglutination test is based on the agglutination of antibody coated cells or particles. Hall and co-workers [19] developed a single-gel diffusion test to determine SEB in custard, chicken, shrimp and other solid foods in 1963. Then, in 1965, double-gel diffusion procedures were reported to assay SEA and SEB in cheese, with respective detection limits as low as 0.02 and 0.05 µg per gram of cheese [20]. In 1968, Salomon and Tew introduced a latex agglutination test for SEB detection [21]. This assay used latex particles coated with specific anti-SEB antibodies as indicators and the limit of detection was 0.2 ng/mL. After that, a latex agglutination inhibition test for SEB detection was developed with a detection limit of 0.5 mg/mL [22]. In addition, reverse passive latex agglutination (RPLA) test kits were developed and applied for SE determinations in pasta, beef, ham, cooked turkey, and some dairy products [23,24]. However, serologic tests are semi quantitative and lack specificity and sensitivity. Thus, they have not been further developed for detection of SEs.

## 3. SE Identification Using Molecular Biological Methods

Molecular biological methods include nucleic acid hybridization and polymerase chain reaction (PCR). Nucleic acid hybridization techniques have been used as an additional means of analyzing strains containing toxin genes by using gene-specific nucleotide sequences as probes. Gemski and co-workers [25] first reported that oligonucleotide probes targeting different enterotoxins genes would allow specific detection and differentiation of toxigenic *S. aureus* isolates by colony blot hybridization. Since then, dot blot hybridization techniques have been developed and applied to determine SE genes in staphylococcal strains [26]. In addition, PCR has been widely used to detect SEs by amplifying corresponding genes [27]. The detection of SEs by PCR was first reported by Wilson *et al.* [28], who used two sets of primers to amplify the SEB and SEC genes (*entB* and *entC1*) and the staphylococcal nuclease gene (*nuc*). This process allowed the detection of *ca.* 1 fg of purified target DNA in dried skimmed milk. Currently, several PCR variants have been developed to detect SEs, such as multiplex PCR [29,30], real-time PCR [31,32], reverse-transcriptase PCR (RT-PCR) [33,34], and loop-mediated isothermal amplification (LAMP) [35,36,37]. Compared to the other PCR-based techniques, the distinct advantage of multiplex PCR is simultaneous detection of several SEs with different primers. PCR also has the advantage of being combined with other techniques, such as most probable number (MPN-PCR) [38], and PCR-enzyme linked immunosorbent assay (PCR-ELISA) [39], which can provide sensitive results. Among the conventional techniques available (compared with animal tests), PCR is much faster and can be applied to detect SEs in most kinds of food, such as vegetable pulav, milk, cheese, and meat products. However, because of interference with target-cell lysis necessary for nucleic acid extraction, nucleic acid degradation and/or direct inhibition of PCR, false negative PCR results may occur. Moreover, routine detection of microbes using PCR can be expensive and complicated, and professional operators are required to perform the assays.

## 4. SE Identification Using Chromatography Methods

SEs are basic proteins that can be enzymatically degraded to peptides, which can be subsequently separated by liquid chromatography (LC), and analyzed by collision-induced dissociation tandem mass spectrometry (MS/MS), yielding information regarding the analyte based on the molecular weights or primary sequences of amino acids. Kientz and co-workers [40] first used liquid chromatography electrospray ionization mass spectrometry (LC-ESI-MS) with TSK gel Phenyl-5PW column packing to detect SEB at concentrations as low as 3 pmol/mL. Since then, there have been several successful reports about quantitative tests for detection of SEs in juices, milk, green beans, crackers, chicken meat, and shrimp by using LC-MS/MS [41,42,43,44,45]. LC-ESI/MS has many advantages over traditional techniques, such as avoiding preliminary steps to isolate the toxins from food, the possibility of quantification and lower limits of detection. However, LC-ESI/MS is usually used for the detection of SEs in food samples with low concentrations of soluble proteins rather than high protein samples, such as milk, since digestion of milk proteins will produce a large number of peptides and suppress electrospray.

## 5. SE Identification Using Immunoassays

Immunoassay is an analytical method that is widely applied in many fields, including pharmaceutical analysis, toxicological analysis, bioanalysis, clinical chemistry, and environmental analysis based on specific recognition between antigens and antibodies. Immunoassays are highly sensitive and specific, rapid to operate, and can be used to detect SEs in complex samples without extensive pre-treatment. Combining antibodies as a recognition component with an appropriate transducer formed biosensors called immunosensors. In immunosensors, sensing elements play an important role in the detection process and are basic devices giving an output quantity in the form of measurable energy that is correlated with the input quantity. According to the applied transduction patterns, these sensors can be classified into three main types: (1) optical detection techniques, (2) electrochemical detection techniques and (3) mass detection techniques.

### 5.1. Optical Immunoassays

**Figure 1:**
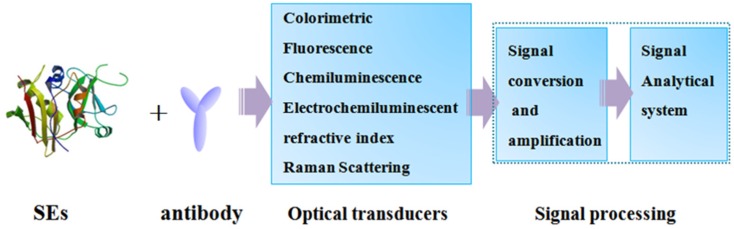
Schematic illustration of optical immunosensors.

Compared with conventional analytical techniques, optical-based detection methods have good application prospects in biomolecular analysis, as they exploit light absorption, fluorescence/luminescence, chemiluminescence, Raman scattering and refractive index for change detection (Figure 1). Optical-based detection methods provide fast, highly sensitive, real-time, and high-frequency monitoring which can make the detection of SEs less time-consuming, lower sample concentration, as well as easier sample pre-treatment. In recent decades, optical-based immunoassays have been widely applied for SE detection in food.

#### 5.1.1. Colorimetric Immunoassays

Colorimetric immunoassays determine analytes by comparing or measuring the absorbance of a colorful substance. The transducer moiety is a key component of colorimetric immunoassays that affects performance with respect to sensitivity, specificity, response time, and the signal-to-noise ratio, due to its function of translating the detecting behavior into light absorption ranging from 390 to 750 nm, characterized by an eye-sensitive color change.

Enzyme-linked immunosorbent assay (ELISA) is a fundamental and widely used colorimetric method. ELISAs are commonly performed by immobilizing artificial antigens or capturing antibodies on plastic supports. The antigen captured by the antibody support can be detected either by an enzyme-labeled antibody specific for the same determinant as the capture antibody or by an enzyme-labeled antibody recognizing a different epitope on the captured, multivalent antigen. Quantification of antigens is achieved by monitoring the cleavage of the chromogenic substrate (e.g., 3,3′,5,5′-tetramethylbenzidine (TMB)) by the enzyme (e.g., horseradish peroxidase (HRP)), which produces a blue metabolite for signal detection. These methods have many advantages, such as being easy to use, specific, and applicable for high throughput screening. SEs are routinely assayed immunologically by ELISA.

Saunders and Bartlett [46] introduced an ELISA for SEA detection by the color reaction of ABTS (2,2′-azino-di(3-ethyl-benzthiazoline-6-sulfonate)) and HRP. The effective monitoring range was from 0.4 ng/mL (20 h detection time) to 3.2 ng/mL (1–3 h detection time). Several ELISA techniques have since been developed for the detection of SEs in culture supernatants and in food samples such as cheese, potato salad, ham, and milk [47,48,49,50,51,52,53,54,55,56,57]. To increase detection sensitivity, alternative methods were developed to maximize signal amplification using a process in which the enzyme-antibody conjugate is formed with a high enzyme to antibody ratio. By incorporating the avidin-biotin system into the ELISA, the sensitivity for detecting SEA and SEB can be improved because there are four binding sites for biotin in one avidin which can amplify the signal [58]. Morissette and co-workers [59] developed a sensitive screening sandwich ELISA for the detection of SEB in cheese using avid polyclonal antibodies to SEB and a biotin-streptavidin amplification system. SEB could be detected in the minimum range from 0.5 to 1.0 ng/mL. Another colorimetric capture ELISA for SEA and SEB was established in test buffer and undiluted human urine with high sensitivity and accuracy. This assay had linear working curves in the range of 0.05 to 1 ng/mL, with a lower detection limit of 0.05 ng/mL in assay buffer and 0.1 ng/mL in human urine using the reaction of substrates (*p*-nitrophenyl phosphate) and neutravidin-linked alkaline phosphatase bound with biotinylated antibody [60]. In summary, ELISA is a common method of SE detection, which can normally be inspected with the naked eye or no instrumentation or a simple portable spectrometer, and is perfect for on-site detection. However, long incubation times and multiple wash steps may lengthen the assay time and it is difficult to apply ELISAs in multi-SEs detection due to the relative one-fold color reaction of chromogenic substrate and enzyme.

#### 5.1.2. Fluorescent Immunoassays

**Figure 2:**
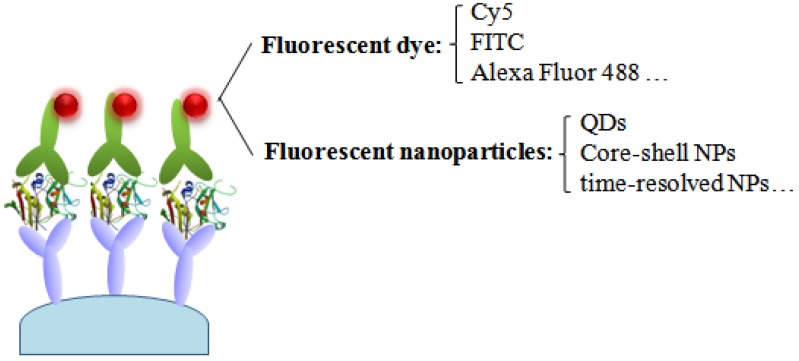
Schematic of a fluorescent immunosensor based on fluorescent dye and fluorescent nanoparticles as labels.

Currently, fluorescent immunoassays are the most popular approach for the detection of SEs. A variety of labels have been used in the development of SE immunoassays including fluorescent dyes or fluorescent nanoparticles producing optical signals that can be correlated with the concentration of the analyte (Figure 2). Compared with enzyme-mediated immunoassays, it was determined that fluorescent-based immunoassays possessed greater potential to provide high-throughput analysis and increased sensitivity.

Tempelman and co-workers [61] first reported a sandwich fluorescent immunoassay for the detection of SEB. In this work, capture antibody was designed as an affinity-purified rabbit anti-SEB, and detection antibody was designed as an affinity-purified sheep anti-SEB labeled with cyanine dye (Cy5). The limit of detection for SEB in PBS buffer is 0.5 ng/mL. Thereafter, Cy5-labeled mouse anti-SEB antibody adsorbed on a membrane of a 96-well plate was used for the detection of SEB. The sensitivity was improved to 0.1 ng/mL [62]. This fluoroimmunoassay is faster due to direct labeling compared with classical ELISA. However, organic fluorophore spectroscopy is characterized by a narrow excitation band and a broad emission band. Additionlly, many organic dyes exhibit low resistance to photodegradation [63]. These characteristics limit their use as effective labels. Luminescent nanoparticles, such as quantum dots (QDs) and lanthanide ion chelate-doped nanoparticles, have been employed to improve optical assay performance via their advantages such as high photostability, quantum yield, and broad absorption with narrow emission spectra. Goldman and co-workers [64] prepared size-dependent tunable photoluminescence CdSe-ZnS core-shell quantum dots (QDs) which possessed narrow emission bandwidths and broad adsorption spectra for the detection of SEB. In addition, they developed a novel conjugation strategy based on electrostatic interactions between negatively charged dihydrolipoic acid (DHLA)-capped CdSe-ZnS core-shell QDs and a positively charged leucine zipper10 interaction domain appended onto the *C*-terminus of engineered recombinant proteins, which was a departure from previously reported conjugation techniques, such as avidin-biotin technology. Vinayaka and Thakur [65] utilized bioconjugated CdTe with immobilized anti-SEB antibody to establish a competitive fluoroimmunoassay for SEB using an immunoreactor column with a limit of detection of 8.15 ng. Later, they developed a fluorescence resonance energy transfer (FRET)-based immunoassay for the detection of SEB [66]. In this work, two tunable photoluminescence cadmium telluride (CdTe) QDs, QD523 and QD601, were synthesized and bound with anti-SEB IgY antibody and SEB, respectively. The specific combination between SEB and anti-SEB antibody resulted in efficient energy transfer between respective QDs, resulting in fluorescence quenching of QD523 and fluorescence enhancement of QD601. FRET is a typical homogeneous assay technique. It could achieve direct detection without a separation step, which makes the experimental procedure simple and efficient.

In addition to QDs, time-resolved fluorescence (TRF) from lanthanide ion chelate-doped nanoparticles is also an excellent label that exhibits good stability against photobleaching, low cytotoxicity, and desirable biocompatibility. There are several immunoassays developed based on TRF for SEB determination [67,68]. The TRF assay can greatly increase signal-to-noise ratio and showed greater sensitivity. Besides, fluorescent dye-doped, core-shell, silica microspheres, such as Alexa Fluor 488 and Rubpy, were also used for SE detection in fluorescent immunoassays [69,70,71]. Although, fluorescence immunoassays have potential advantages for simultaneous detection of multiple SEs due to multi-dyes and fluorescent nanoparticles as labels, there are few reports about the simultaneous detection of multiple SEs. Rubina and co-workers [72] developed an approach to the simultaneous determination of Staphylococcal enterotoxins A, B, C1, D, E, G, and I in a diagnostic detection system.

#### 5.1.3. Chemiluminescence Immunoassays

Chemiluminescence (CL) is also a promising analytical technology with high sensitivity, wide linear range, simple instrumentation, and no background scattering light interference. CL-based immunoassays have been widely used for SE determination. HRP is a commonly used regent in CL detection with the luminol-H_2_O_2_-HRP-PIP (*p*-iodophenol)-enhanced CL system. [73,74]. The Jin group [75] used a pair of highly specific mAbs as well as a highly sensitive chemiluminescent substrate for the HRP-luminol-H_2_O_2_ system and established a microplate chemiluminescent enzyme immunoassay. This work greatly enhanced the detection sensitivity with a limit of detection (LOD) of 0.01 ng/mL. Moreover, good feasibility was achieved by applying this CL-based immunoassay to roast beef, peanut butter, cured ham, milk, and orange juice. Subsequently, a similar strategy was used for SEA detection, which exhibited high performance, with a wider working curve range from 6.4 pg/mL to 1.6 ng/mL and a lower LOD of 3.2 pg/mL [76]. To enhance sensitivity, poly (horseradish peroxidase) (PolyHRP40) was used as a label instead of HRP to develop a CL immunoassay for SEB detection [77]. The chemiluminescence signal was amplified using PolyHRP40, which is a super-molecular complex composed of five identical covalent HRP homopolymer blocks covalently coupled to streptavidin molecules, resulting in a large number of signal-generating enzyme molecules per one bound analyte molecule. The Zhang group [78] developed a novel CL immunoassay using HRP functionalized mesoporous silica nanoparticles (MSNs) as the label. The highlight of this assay is a signal amplification process, where a large amount of HRP was introduced onto the MSN carriers to maximize the ratio of enzyme per sandwich immunoassay. The sensitivity was improved to as low as 4 pg/mL. Prior to this, the research group coupled CL with a charge-coupled device (CCD) imaging detector to establish a method for SEC_1_ detection. CCD detectors can be exposed to CL for long periods of time and obtain low background noise, thus making signal to background ratios high. Benefitting from the advantages of CL imaging, SEC1 was detected by this powerful detection system with a detection limit of 0.5 ng/mL [73].

Nanoparticles have the potential to enhance the sensitivity of biodetection because of their large surface area and electrical conductivity. Recently, attention has been paid to a novel class of newly discovered nanocarbons, that is, carbon nanotubes (CNTs) because of their electronic properties combined with a large specific surface area for antibody immobilization and low absorption in the visible range. Several studies on CNT applications have been conducted by the Rasooly group. They reported a CNT-based CL immunoassay for SEB detection in soy milk, apple juice and baby food [79,80]. In their reports, they utilized CNT to immobilize primary antibody and enhanced CL signal by CCD for detection. In this system, anti-SEB primary antibodies were fixed onto the CNT surface by electrostatic adsorption, and then the antibody–CNT mixture was fixed onto a polycarbonate surface. The SEB was then detected by a “sandwich-type” CL immunoassay on the CNT-polycarbonate surface. This combination achieved highly sensitive detection levels with relatively simple and inexpensive equipment. Although effective, the CNTs were toxic and difficult to manipulate. Therefore, other types of nanoparticles were investigated with the purpose of enhancing the sensitivity of the SEB immunoassays detection. The surface area and geometric and physical properties of gold nanoparticles make them well-suited for enhancing interactions with biological molecules in assays. The Rasooly group established a gold nanoparticle-based CL immunosensor for the detection of SEB using a CCD detector with a detection limit of 0.01 ng/mL [81]. This developed method was also applied to real food samples, such as mushrooms, tomatoes, and baby food meat. They concluded that gold nanoparticles are generally preferable because of their lower toxicity and easier immobilization of antibodies than CNTs. In addition to the luminol-H_2_O_2_-HRP-PIP system, a bis(2,4,6-trichlorop henyl) oxalate (TCPO)-H_2_O_2_-glyoxaline-PHPPA dimer chemiluminescent system was also used to detect SEB [82]. This method showed a detection limit of 3.3 pg/mL and was successfully applied in the analysis of serum, lake water and milk samples.

Compared with fluorescence and UV–visible spectrophotometry, CL-based immunoassays have the advantages of simple instrumentation, low cost, and easy automation due to the lack of an excitation source and optical filters.

#### 5.1.4. Electrochemiluminescence Immunoassays

Electrochemiluminescence (ECL) is a means of converting electrochemical energy into radiative energy at the surface of an electrode through an applied potential. Luminescence signals can be obtained from the excited states of an ECL luminophore generated at the electrode surface during the electrochemical reaction. Considerable attention was focused on tris(2,2′-bipyridyl)ruthenium(II) (Ru(bpy)_3_^2+^) (including its analogues) and its coreactants in the ECL system. ECL biosensors possess the advantages of rapidity and high sensitivity, a simplified set-up, low background signal and ease of miniaturization and have thus received considerable attention from many researchers in recent decades. [83,84]. However, there are few reports of applying ECL to SE detection.

Kijek and co-workers first developed an ECL system for the detection of SEB using capture antibody-coated magnetic beads and detector antibody conjugated ruthenium (II) tris-bipyridal (Ru(bpy)_3_^2+^) [85]. By virtue of immunomagnetic separation and the high luminescent signal-to-noise ratios of ECL, SEB can be reproducibly detected as low as 1 pg/mL in serum, urine, tissue or buffer samples. ECL could also be combined with ELISA-lab-on-a-chip (LOC) for the immunological detection of SEB [86]. A charge-coupled device detector was used to measure the light signal of ECL for detection of SEB and achieved a detection limit of 0.1 ng/mL. To simplify the experimental procedures and to reduce testing costs, an automated point-of-care (POC) immunodetection system, combining the ELISA-LOC format with a sensitive ECL detector, was designed and fabricated for SEB determination [87]. This POC system can be used for a variety of targets without dedicated laboratories and complex equipment.

#### 5.1.5. Surface Plasmon Resonance (SPR) Immunoassays

Surface plasmon resonance (SPR) is one of the most exciting surface-sensitive methods for SE detection. In an SPR immunoassay, antibodies were coated onto the sensor chip and the reflection intensity changed when the target bound with the antibodies. The refractive index changes were realized through the vicinity of thin metal layers (i.e., gold, silver, or aluminum films) in response to interactions between antibodies and targets. SPR features surface-sensitive response, label-free detection, and real-time measurement capability. Therefore, the presence of the analyte can be determined directly without the use of labeled molecules, which makes continuous real-time detection of biomolecular analytes possible.

Numerous studies have applied SPR to SE detection. The Rasooly group [88] introduced a sandwich SPR immunoassay to monitor SEA in milk, hotdogs, mushrooms and potato salad with a detection limit of 10–100 ng/g. In this sandwich-type SPR immunosensor, two antibodies were used. The capturing antibody was fixed on the surface of the biosensor chip to bind with the antigen, and a second antibody binds to the captured antigen. The second antibody not only makes antigen verification possible but also amplifies the signal. The Rasooly group also utilized the sandwich SPR immunosensor to detect SEB spiked in potted meat at 10–1000 ng/g and with a minimum SEB detection of 10 ppb [89]. Another sandwich immunoassay using SPR was also used for the determination of 2.5 ppb SEB-spiked ham tissue [90]. Moreover, a competitive pattern of SPR biosensor for detecting SEA and SEB in whole egg was established with the concentration range of 1–40 ng/mL SEA [91] and the LOD of SEB was lower than 1 ng/mL in fresh fluid milk [92]. SPR immunoassay combined mass spectrometry was established to detect SEB in spiked milk and mushrooms, and achieved a detection limit of 1 ng/mL [93,94]. To enhance sensitivity, Homola and co-workers developed a dual-channel SPR immunoassay based on wavelength modulation and achieved the detection of 5 ng/mL of SEB [95]. Gupta and co-workers developed an SPR immunoassay for SEB determination utilizing a carboxymethyldextran-modified gold chip [96]. This SPR biosensor displayed good linearity from 2.0 to 32.0 pM, with a detection limit of 1.0 pM. SPR immunosensor based on antibody-coated super paramagnetic nanobeads was used for SEB detection [97]. With the help of immunomagnetic beads, the separation and concentration of SEB in analyte-spiked samples became easier, and the SPR detection signal was significantly amplified, making it easy to detect SEB in both buffer and stool samples at a concentration of 100 pg/mL.

#### 5.1.6. Surface-Enhanced Raman Scattering (SERS)-Based Immunoassay

SERS(Surface-enhanced Raman scattering), a Raman spectroscopy technique that is capable of obtaining species fingerprint information by detecting vibrational bands, has been used for qualitative and quantitative analyses, giving a rapid, reliable, and sensitive result. However, SERS-based immunoassays for SE detection have not been popular. Pekdemir and co-workers reported a SERS-based immunoassay for the detection of SEB by polyclonal-antibody functionalized magnetic gold nanorod particles as capture probes, and 5,5-dithiobis (2-nitrobenzoic acid) (DTNB) on gold nanoparticles as a Raman-active reporter molecule [98]. The detection limit for the assay was 768 aM. SERS-based immunoassays should be expanded to SE detection due to their wide linear range, good sensitivity, operational simplicity, and short assay time.

### 5.2. Electrochemical Immunoassays

Electrochemical immunoassays are another possible method for the quantification of SEs based on the change in an electric signal. The electric signal can be observed as a current, potential, impedance or conductance, which may be classified as amperometric, potentiometric, impedimetric and conductometric sensors. Electrochemical immunoassays are simple, sensitive, portable, cheap, and reproducible, having outstanding compatibility with the latest technology. Recently, electrochemical detection methods of SEs based on impedance and current were reported by various researchers.

Dong and co-authors conducted an electrochemical immunoassay for the detection of SEC1 using cyclic voltammetry and AC impedance measurements, and the LOD was 1 mg/mL [99]. An amperometric immunosensor was developed for the detection of SEA based on a sandwich assay. The sensitivity was as low as 33.9 ng/mL. Chatrathi *et al.* [100] developed a sandwich electrochemical immunoassay for the detection of SEB. This sandwich assay consisted of thiolated antibodies immobilized onto the electrode and a second antibody of alkaline phosphatase (ALP)-tagged anti-SEB. With the addition of α-naphthyl phosphate (α-NPP), the ALP tag caused the biocatalytic conversion of non-electroactive substance, α-NPP, into the electroactive substance, α-NP. The detection limit estimated from a representative dose-response curve was 1 ng/mL.

To achieve high sensitivity, different labeling methods and technologies have been employed for the signal amplification of the antigen–antibody interaction. Mishra and co-authors [101] reported a sensitive electrochemical immunoassay for the detection of SEB using polysilicon nanowires for signal amplification. The detection limit was observed in the range of 10–35 fM. Based on horseradish peroxidase-nanosilica-doped multiwalled carbon nanotubes (HRPSiCNTs) for signal amplification, a novel sandwich-type electrochemical immunoassay was designed for detecting SEB [102]. The antibodies of rabbit polyclonal anti-SEB were immobilized on screen-printed carbon electrodes (SPCE) and covalently targeted to the HRPSiCNTs, and then they were used as capture antibodies and detection antibodies, respectively. SEB can be detected at concentrations as low as 10 pg/mL. Wu *et al.* established an electrochemical immunosensor based on a gold electrode modified with a composite film of magnetosomes/1,2-dimethyl-3-butylimidazolium hexafluorophosphate ([D(n-C4)Im][PF6])/polyaniline nano-gold composite (PANI/Au) for the detection of SEB [103]. With the larger surface area offered by the magnetosomes, enhanced response signals of PANI/Au, and good conductivity and stable electrochemical response provided by [D(n-C4)Im][PF6], the immunosensor showed a detection limit as low as 0.017 ng/mL. Furthermore, the developed method was successfully applied to milk sample detection with high recoveries from 81% to 118%.

### 5.3. Mass-Based Immunoassays

Mass-based immunoassays are another sensitive detection method, in which transduction is based on small changes in mass. The basic means of mass analysis depends on the use of piezoelectric crystals and amorphous ferromagnetic ribbons or wires. Piezoelectric (PZ) devices consist of an oscillating quartz crystal with an adsorbent on its surface with selectivity toward the analyte. The adsorption of the analyte increases the mass of the crystal and proportionally decreases the resonance frequency of oscillation. Harteveld and co-workers [104] introduced a piezoelectric crystal immunosensor for SEB detection based on a competition scheme using polyclonal antibodies (anti-SEB), and a LOD of 0.1 µg/mL was obtained. Later, a reusable PZ immunosensor was developed for the detection of SEC_2_ by coating SEC_2_ antibody on PZ crystals [105]. The immunosensor could be reused six times. In addition, a quartz crystal microbalance biosensor using sol-gel imprinting was developed for SE detection [106]. Moreover, Mutharasan’s group [107,108] reported a highly sensitive and rapid method for the detection of SEB using a piezoelectric-excited, millimeter-sized cantilever (PEMC) sensor by immobilizing antibody on the animated cantilever glass surface. The detection of SEB was accomplished by monitoring the sensor’s fundamental resonance frequency. In addition, there are similar piezoelectric immunosensors for the detection of SEB [109] and SEA [110,111]. Magnetoelastic sensors are analogous to piezoelectric acoustic wave sensors, which measure parameters of interest by tracking the changes in their resonance behavior. The basic operating principle of these sensors involves a change in sensor resonance frequency because of mass loading, which is related to the binding of an analyte to a receptor that immobilized on the surface of the ribbon-like magnetoelastic sensor. Grimes and co-authors [112] described a magnetoelastic immunosensor for the detection of SEB with affinity-purified rabbit anti-SEB antibody covalently immobilized on magnetoelastic sensors. With the use of alkaline phosphatase as the labeled enzyme to induce biocatalytic precipitation, the biosensor demonstrated a detection limit of 0.5 ng/mL. The most important advantages of piezoelectric and magnetoelastic sensors are the high sensitivity for detection and the absence of the necessity to introduce additional labels, which reduce the response time and the cost.

Although immunobioassays are a reliable and technology for SE detection, which exhibit high sensitivity, wide linear range and feasibility, they require high quality antibodies. The preparation of the antibodies via animal immunization experiments is tedious and time-consuming. Besides, the obtained antibodies may be unstable, and susceptible to modification issues.

## 6. New Trends

### 6.1. Aptamer-Based Bioassays

To rival antibodies, aptamers with high affinity and selectivity are starting to emerge. Aptamers are short ssDNA or RNA molecules that were selected through *in vitro* selection or the systematic evolution of ligands by exponential enrichment (SELEX) [113,114]. Aptamers bind to target molecules with similar affinity and specificity as antibodies, but they possess many competitive advantages over antibodies. [115]. Aptamers can be easily produced by chemical synthesis at higher purity, and flexibly modified with various chemical tags that do not influence the affinity. Moreover, aptamers have small molecular weights and superior stability that can bear repetitious denaturation and renaturation. Overall, these unique characteristics make aptamers an ideal recognition element for biosensors. Recently, aptamers have been alternative recognition elements applied to SEs detection.

Bruno and Kiel [116] first selected aptamers targeted to SEB using magnetic bead-based SELEX. Affinity selection of aptamers was accomplished by conjugating the SEB to tosyl-activated magnetic beads and five rounds of SELEX. Unfortunately, the specificity of the SEB aptamer was not studied, and its sequence has not been revealed, which severely limits its wide applications in public health protection. A decade later, DeGrasse [117] reported an aptamer sequence that selectively binds to SEB based on magnetic bead-SELEX and aptamer-precipitation experiments. The aptamer APT^SEB^ was characterized to bind to SEB with high selectivity amongst other SEs. Liu *et al.* [118] obtained a panel of aptamers against SEB by optimizing the procedure of SELEX. Then, an unmodified AuNPs-based colorimetric method employing the selected aptamers was developed to detect SEB, and the detection limit was 10 ng/mL. DeGrasse and Liu’s research has raised the possibility of the application of aptamers in SE determination and promoted their development. By employing the aptamers as recognition elements, piezoresistive cantilever-based aptasensors were developed for SEB detection both in buffer and skim milk [119]. The results demonstrate that piezoresistive cantilever-based aptasensors can detect SEB in food with sufficient sensitivity and build a foundation for the development of on-site SE detection systems.

**Figure 3:**
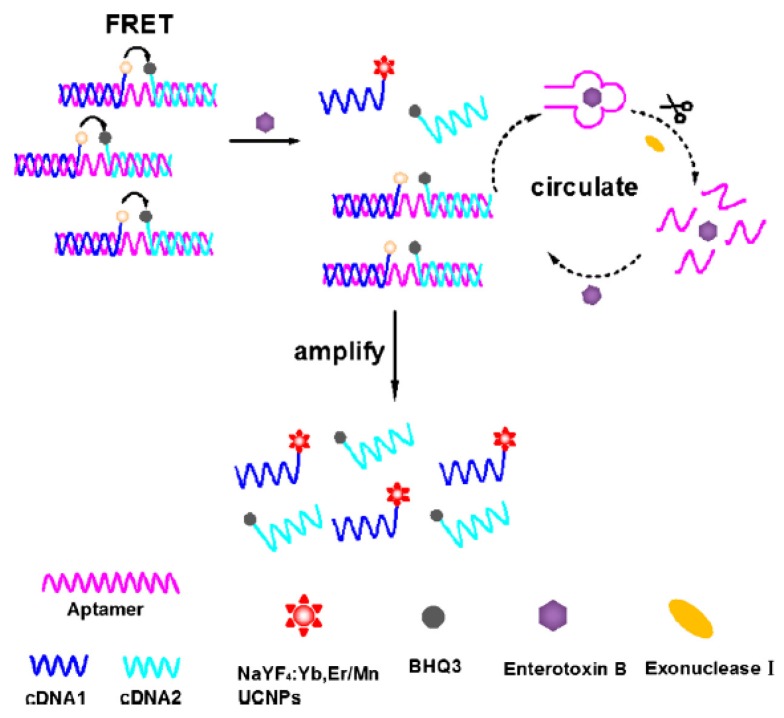
Schematic illustration of an exonuclease-catalyzed target recycling strategy for SEB detection based on FRET. (Adapted with permission from reference [120] Copyright 2013. Elsevier).

The Wang group developed an FRET bioassay to detect SEB by using an aptamer-affinity method coupled with upconversion nanoparticles (UCNPs) sensing, and the fluorescence intensity was prominently enhanced using an exonuclease-catalyzed target recycling strategy [120]. In this aptasensor, both fluorescence donor probes (cDNA 1-UCNPs) and fluorescence quencher probes (cDNA 2-Black Hole Quencher 3 (BHQ_3_)) were hybridized to an SEB aptamer to form a duplex, which quenched the fluorescence of the UCNPs via FRET (Figure 3). The formation of an aptamer-SEB complex resulted in the dissociation of aptamer from the duplex and the restoration of the UCNPs fluorescence. Exonuclease I selectively degraded the single-stranded DNA (ssDNA) in the aptamer–SEB complex, with the digestion finishing after the ssDNA was fully consumed; however, the dsDNA was not digested by the solution. In this way, SEB was released and could change the aptamer conformation from the dsDNA to the aptamer–SEB complex, which thereby amplified the fluorescence of the UCNPs in the circulatory system. With the superior optical features of the UCNPs, the high affinity and specificity of SEB aptamers, and the introduction of exonuclease I into the analyte recycling system, excellent sensitivity (LOD of 0.3 pg/mL) was obtained using this protocol. Moreover, the Wang group enlarged the use of aptamer-based bioassay application in SE detection by selecting aptamers targeted to SEA and SEC_1_, based on magnetic separation technology [121,122]. Combining the SEB aptamer selected by DeGrasse, the Wang group established the simultaneous detection of multiplex SEs (SEA, SEB and SEC1) using multicolor lanthanide-doped TRF labeled aptamers as bioprobes and graphene oxide (GO) as a resonance energy acceptor [123]. This is the first report of aptasensor application in the simultaneous detection of multiple SEs with the advantages of high-throughput, sensitivity, and convenient performances. This work created a new technology for SE detection and was significant in the development of methods to improve food safety.

**Figure 4:**
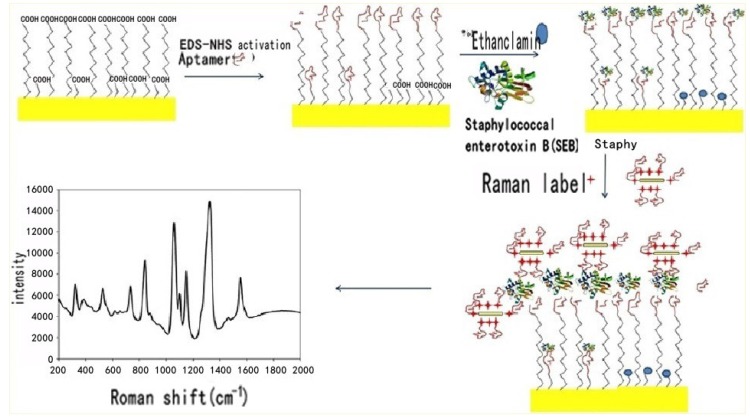
Schematic illustration of peptide-based SERS sensor and SPR sensor system for detection of SEB. (Adapted with permission from reference [127] Copyright 2012. American Chemical Society).

Apart from ssDNA aptamers, peptide aptamers, especially those derived from phage-displayed peptide libraries, are becoming popular as novel recognition agents in bioassay and biosensor applications. The Boyaci research group conducted a comprehensive and in-depth study on peptide aptamers selection and the application to SEs. They first used a peptide-phage display library to assess affinity of peptides for SEB [124]. The dissociation constant of the selected peptide was 4.2 ± 0.7 × 10^5^ mol/L which shows strong binding ability. This study suggested that the peptides selected by rational approaches with improved affinity can be used in biosensors instead of antibodies. However, this study did not involve an examination of the mechanism of the peptide–SEB interaction. Two years later, Dudak *et al.* reported a thermodynamic and structural analysis of the binding interactions between peptide ligands and SEB [125]. They studied the core amino acids playing an important role in the binding between the peptides and SEB. The approach to the thermodynamic and structural characterization of peptide ligands can be used to develop aptamers with high affinity and selectivity for SEB biosensor applications. Moreover, to improve the binding ability of the SEB peptide aptamers, Dudak *et al.* explored new modification approaches by repeating the 12-mer peptide sequences [126]. Based on these studies, they developed a SERS biosensor [127] (Figure 4) and a SPR biosensor [128] to demonstrate the potential application of peptide aptamers for SEB detection in a complex matrix.

### 6.2. Molecularly Imprinted Polymers (MIPs)-Based Bioassay

Molecularly imprinted polymers (MIPs) embody a new class of materials possessing high selectivity and affinity for the target molecules. MIPs possess molecular recognition properties due to the cavities produced in the polymer matrix that are complementary in size and shape to the target imprint molecule [129]. Compared with proteins and nucleic acids, MIPs are more stable and can resist much harsher conditions, such as high temperature, extreme pH, pressure and organic solvents. In addition, MIPs preparation does not require animals, which are essential for antibody production. MIPs are stable and economical sensing elements and are suitable for the substitution of natural receptors and antibodies in assays.

**Figure 5:**
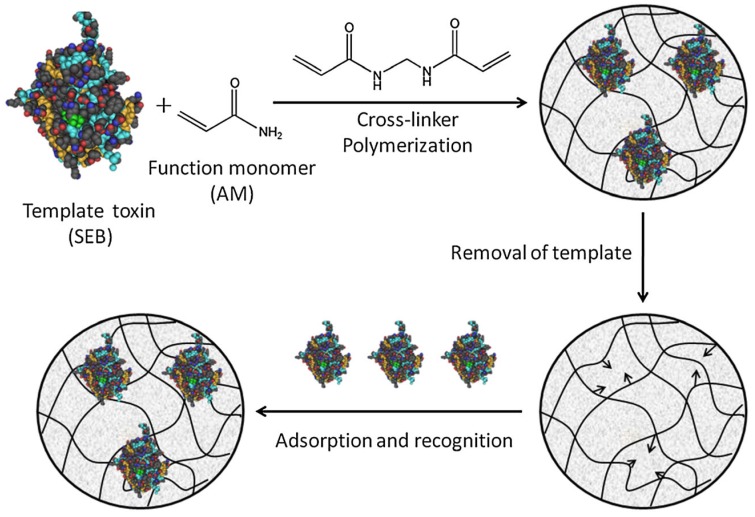
Schematic representation of protein toxin imprinting and illustration of the binding process. (Adapted with permission from reference [131] Copyright 2014. Wiley-Blackwell).

Recently, several methods have been developed for SE determination based on MIPs as the recognition molecules for SEs. Gupta and co-workers [130] reported SPR biosensor for the detection of SEB by employing MIPs as the sensing material. In this assay, MIP film was prepared by *in situ* electropolymerization of 3-aminophenylboronic-acid (3-APBA) on the bare gold chip. In the presence of SEB, the MIP-based biosensor generates a signal by specific molecular interaction between the MIPs and SEB with a detection limit of 0.05 fM. In addition, protein-imprinted polyacrylamide gel beads (IPGB) were also synthesized by using SEB as the template molecule based on inverse suspension polymerization [131] (Figure 5). The maximum adsorption capacity of SEB-IPGB can achieve 8.40 mg SEB/g imprinted beads. MIPs have potential applications in SEB separation, extraction, and detection as economical molecule recognition materials.

The Gao group synthesized a two-dimensional (2D) molecularly imprinted sol-gel thin film-coated quartz crystal microbalance (QCM) that immobilized on the surface of a piezoelectric quartz crystal (PQC) Au-electrode for the detection of SEB [132]. The LOD was 6.1 ng/mL, and the detection period was within 0.5 h. This method is simple, fast, sensitive, and low cost. Based on the former research, they firstly apply the QCM sensor in the simultaneous detection of SEA and SEB based on sol-gel thin film imprinting technology with the lower detection limits of 7.97 and 2.25 ng/mL, respectively [133].

## 7. Summary and Future Prospects

Conventional SEs detection methods, such as animal tests and serologic tests, are labor-intensive and unable to meet the needs of real-time detection. PCR-based molecular biological analysis is a reliable and much faster method with conclusive results. However, PCR-based methods are aimed at DNA analysis, not for the SEs themselves, and are therefore indirect detection methods. The chromatographic methods are efficient and sensitive, but the requirements of tedious sample pre-treatment steps, expensive instruments and trained personnel limit its application in on-site measurements. New technologies, such as immunosensors combined with optical, electrical and mass transducer detection techniques, are reliable options. The developing bioassays based on new recognition elements, such as aptamers, peptides, and MIPs, for SE detection have superior characteristics of sensitivity, selectivity, rapidity and reliability. Especially, aptamers bind target molecules with affinity and specificity equal or superior to antibodies, and they are easily synthesized and modified and more stable. The utility of aptamers improves biosensor characteristics and expense, demonstrating that the aptasensors are adequate for practical use; and they also are rapid, reliable, effective and suitable for *in situ* analysis. In the future, the selection of aptamers to target SEs should be amplified and biosensors based on aptamers for SE detection should also be developed. Moreover, the research into detection methods for SEs will focus on progressing simultaneous multi-element analysis. Miniaturized, portable and easily disposable commercial applications facilitate testing on site without a laboratory and point of care detection of SEs.
